# VIRTUAL REALITY TO SUPPORT THE NEEDS OF OLDER ADULTS WITH AND WITHOUT COGNITIVE IMPAIRMENTS

**DOI:** 10.1093/geroni/igad104.0318

**Published:** 2023-12-21

**Authors:** Walter Boot

**Affiliations:** Florida State University, Tallahassee, Florida, United States

## Abstract

Functional, cognitive, and mobility limitations can interfere with older adults’ ability to engage in socially and cognitively enriching activities, which can lead to further decline. Emerging technology solutions, including virtual reality (VR), have the potential to promote engagement in these activities in a way that can account for these limitations, allowing older adults to engage in productive and meaningful activities within their own home. However, an iterative, user-centered design process is necessary to ensure that these solutions reach their potential. This talk provides an overview of a number of recent studies examining the potential of VR to support older adults’ engagement in VR interventions aimed at improving mood and social connectivity. The following questions will be asked and answered during this talk, based on recently collected empirical data; What are the potential usability challenges associated with VR interventions?; What are older adults’ preferences for different VR experiences?; Can VR provide older adults with meaningful opportunities to engage in social interactions when they are distally located, but co-located in the same virtual space?; To what degree do older adults feel present in the virtual environment?; To what degree are older adults’ attitudes toward VR malleable?; If, and under what conditions, is cybersickness a potential barrier? This talk will conclude with a discussion of what is known and what is unknown regarding the use of VR to provide meaningful socially and cognitively engaging experiences for older adults with and without mild cognitive impairment.

